# A Sperm Quality Detection System Based on Microfluidic Chip and Micro-Imaging System

**DOI:** 10.3389/fvets.2022.916861

**Published:** 2022-06-30

**Authors:** Xiaoqing Pan, Kang Gao, Ning Yang, Yafei Wang, Xiaodong Zhang, Le Shao, Pin Zhai, Feng Qin, Xia Zhang, Jian Li, Xinglong Wang, Jie Yang

**Affiliations:** ^1^Institute of Animal Science, Jiangsu Academy of Agricultural Sciences, Nanjing, China; ^2^School of Electrical and Information Engineering, Jiangsu University, Zhenjiang, China; ^3^School of Agricultural Engineering, Jiangsu University, Zhenjiang, China; ^4^College of Animal Science and Technology, Yangzhou University, Yangzhou, China

**Keywords:** sperm quality, microfluidic chip, micro-imaging system, motility, survival rate

## Abstract

Sperm quality assessment is the main method to predict the reproductive ability of livestock. The detection of sperm quality of livestock is of great significance to the application of artificial insemination and *in vitro* fertilization. In order to comprehensively evaluate sperm quality and improve the real-time and portability of sperm quality detection, a portable microscopic imaging system based on microfluidic chip is developed in this paper. The system can realize the comprehensive evaluation of sperm quality by detecting sperm vitality and survival rate. On the hardware side, a microfluidic chip is designed, which can automatically mix samples. A set of optical system with a magnification of 400 times was developed for microscopic observation of sperm. In the aspect of software, aiming at the comprehensive evaluation of sperm quality based on OpenCV, a set of algorithms for identifying sperm motility and survival rate is proposed. The accuracy of the system in detecting sperm survival rate is 94.0%, and the error rate is 0.6%. The evaluation results of sperm motility are consistent with those of computer-aided sperm analysis (CASA). The system's identification time is 9 s. Therefore, the system is absolutely suitable for sperm quality detection.

## Introduction

The main role of sperm is to send the paternal gene into the oocyte during fertilization (1). It plays an important role in the process of gestating life. The sperm quality directly affects the fertilization rate (2). In animal husbandry, accurate prediction of male fertility has great economic significance (3). Screening out males with high sperm quality for reproduction is conducive to expanding the scale of breeding and improving economic benefits. Since 2018, African swine fever has spread widely in China, causing a great impact on the pig industry (4). At present, the epidemic situation of swine fever in Africa has been greatly alleviated (5). Against this background, it is very important to develop a system that can quickly detect the quality of sperm.

In animal husbandry, the methods for estimating sperm quality are mostly combined detection of several indicators in order to accurately reflect the sperm quality and the ability of fertilization (6, 7). Yuki et al. (8) found that there were significant correlations between fertilization rates and computer-aided sperm analysis (CASA) estimates, including amplitude of lateral head displacement (ALH; *r* = 0.269), curvilinear velocity (VCL; *r* = 0.297), straight line velocity (VSL; *r* = 0.266), and rapid sperm movement (Rapid; *r* = 0.243). Viudes-de-Castro et al. (9) used total motility, viability rate and acrosome integrity as rabbit sperm quality variables. Marcus-Braun et al. (10) used cell concentration, percent of motility, and quality of motility to do sperm analysis. Generally speaking, a single index can't accurately describe sperm quality. Only the joint estimation of several indicators will accurately reflect sperm quality.

According to the World Health Organization (WHO), one of the most important attributes in evaluating sperm quality is sperm motility (11). For the detection of sperm motility, traditional methods are conventional sperm analysis (RSA) and CASA. At present, the routine sperm analysis is carried out by experienced medical staff under microscope, which has some disadvantages such as low accuracy and lack of objective data support (12). Compared with the traditional sperm motility evaluation, CASA has a higher added value in artificial insemination of pigs (13). Su et al. (14) used high-throughput lens-free to get the 3D tracking of human sperm. But it can not distinguish sperm with broken membranes. Sperm motility and membrane integrity are both important standards to measure sperm quality, because the integrity of sperm membranes is necessary for sperm to maintain its function during storage and penetration of oocytes in the female reproductive tract (15). Here, the sperm with broken membranes are considered dead sperm, and the dead sperm include inactive sperm and motile sperm with damaged membranes. In addition, it is necessary to estimate the survival rate of the sperm for comprehensive estimation. For the detection of sperm viability, traditional methods include lipid peroxidation of sperm membrane, flow cytometry (FCM) and staining. Juarez et al. (16) used flow cytometry to detect the sperm survival rate of epididymal alpaca sperm during cryopreservation, which has the advantages of rapidity, objectivity and multiple indicators. However, flow cytometry is still in the laboratory stage, and flow cytometry Counters are expensive and difficult to use in practical production. Ebrahimi et al. (17) used bull sperm stained with eosin-aniline black as a control group to verify the reliability of plant dye staining. The background of eosin-aniline black staining is dark purple, so it is easy to observe. But this method requires smears to dry, which means the corresponding increasement in the steps. Generally speaking, the detection of sperm quality relies on complex and expensive instruments or cumbersome biochemical detection methods, which are mostly used in hospitals or laboratories, limiting the application of on-site rapid detection.

Computer-aided sperm analysis often needs a special sperm quality analyzer to complete it, which is inconvenient to carry. In contrast, the application prospect of portable devices is better. Kobori et al. (18) designed a single ball microscope for male infertility detecting. They only paid attention to the number of sperm and their ability to move. However, the decrease of sperm survival rate is also an important factor in male infertility (19). Ashok et al. (20) designed a smartphone-based home sperm detection kit to measure the concentration of moving sperm. But the concentration of moving sperm does not accurately reflect sperm motility, as sperm can be divided into different motility levels (21). Only sperm with high motility have physiological significance. Ilhan et al. (22) developed a sperm counting system based on smart phone is developed, which can be used as an alternative method for sperm concentration analysis in visual evaluation technology. However, none of them are equipped with microfluidic chips, which leads to complicated operation steps and a large number of reagents, making them unsuitable for on-site testing. As for microfluidic chip technology, many researchers have made in-depth discussions on the design of the chips. Chen et al. (23) designed an osmotic phase conduction structure in the microfluidic device, which can easily analyze sperm quality. Eamer et al. (24) used the medium layer designed in the microfluidic device to screen out sperm with strong motility. Whether a paper chip (25) or a PDMS chip, by designing different functional structures (26), the automatic processing of samples can be realized, which reduces many experimental steps, and is very portable (27). Therefore, microfluidic chips play an important role in the fields of medical diagnosis.

In this paper, a self-priming micro-fluidic chip is designed, which can detect sperm motility and survival rate simultaneously. It can automatically inject samples and fully mix the samples at the same time, which simplifies the operation steps. Additionally, a portable micro-imaging device is invented for obtaining microscopic images of sperm samples. Meanwhile, a set of image processing algorithms which can recognize sperm motility and survival rate is proposed to realize the comprehensive estimation of sperm quality. This system has high resolution and portability. It can be used for real-time detection and comprehensive evaluation of pig sperm quality, and to some extent, it can improve the efficiency and reliability of breeding pig screening.

## Materials and Methods

### Sample Preparation

In order to comprehensively estimate the sperm quality, pig sperm samples and eosin-aniline black are prepared respectively. The pig sperm samples come from China Subu Biotechnology Co., Ltd. (Shanghai, China). The boar breed is Dabai with 18 months old.The semen collection season is April in the spring, and a total of 10 boars were collected and mixed.It comes from the SPF pig herd of the national core breeding farm, which ensures the high quality of pig sperm. To facilitate sperm observation, sperm samples are diluted 10 times with 0.9% normal saline at the tem-perature of 37 degrees Celsius. Eosin-aniline black comes from Shanghai Ruichu Biotechnology Co., Ltd. (Shanghai, China), used to identify the integrity of the sperm membrane structure and assess sperm survival rate. In order to facilitate the experiment, we reduced the activity of sperm.

### Design of Self-Priming Microfluidic Chip

The proposed self-priming microfluidic chip is fabricated using standard soft lithography (28). The chip can achieve sample self-priming and sufficient mixing function, which is made of glass and PDMS. For the sample self-priming, a hydrophilic material (29) (Mesophilic-2000, PEG) is injected into the microchannel. After staying for 1 min, the liquid in the microchannel is slowly discharged with air using a pipette gun (100–1,000 μl). Then hydrophilic nano coating is formed inside the microchannel.

**Figure 1:**
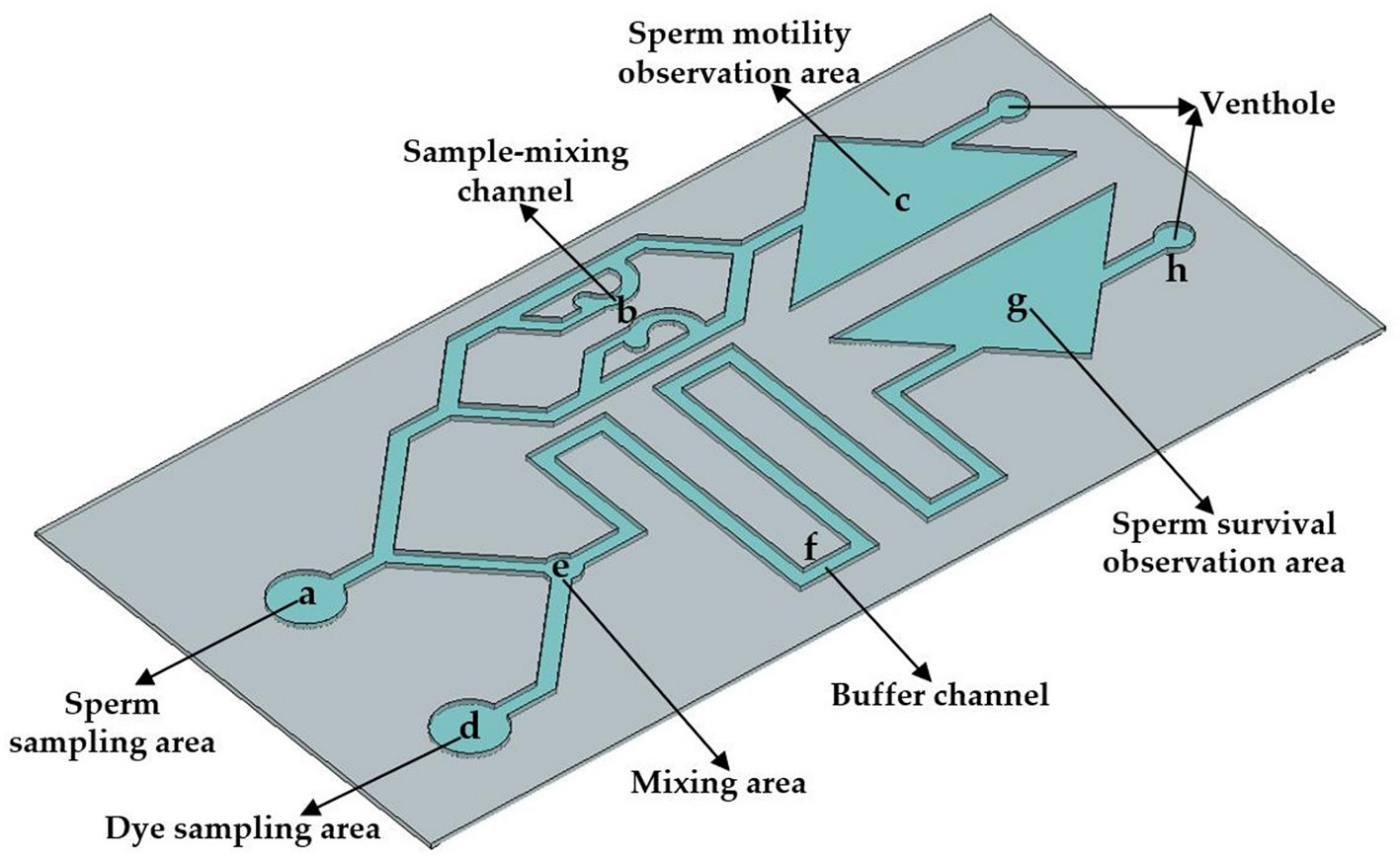
Structure design of microfluidic chip.

In order to complete the evaluation of the quality of pig sperm within 30 s, the size of the microfluidic chip designed in this study is 3 × 6 cm. The height of microfluidic chip channel is 90 μm. The chip is divided into two parts in structure, which can detect sperm motility and survival rate simultaneously. Comprises an upper sperm sampling area, a sample mixing channel and sperm movement observation area. The lower part is consistes of the dye sampling area, mixing area, buffer channel and sperm survival observation area. In the upper part, the sperm sample flows through the sample mixing channel and finally flows into the sperm movement observation area. In the lower part, the dye is mixed with the sperm sample in mixing area and flows through the buffer channel, finally flowing into the sperm survival observation area. Through the design of two observation areas, the chip can detect sperm motility and survival rate at the same time, so as to realize the comprehensive evaluation of sperm quality. As shown in Figure 1, a is the sperm sampling area (the diameter is 4 mm); the sample-mixing channel b is used for the uniform diffusion of the sperm sample; c is the sperm motility observation area; d is the dye sampling area (the diameter is 4 mm); e is the mixing area (the diameter is 2 mm), which is used to fully mix the sperm sample and the staining agent; the buffer channel f is used to fully stain the sperm sample (the width is 1 mm); and g is the sperm survival observation area.

### Portable Micro-Imaging Recognition System

**Figure 2:**
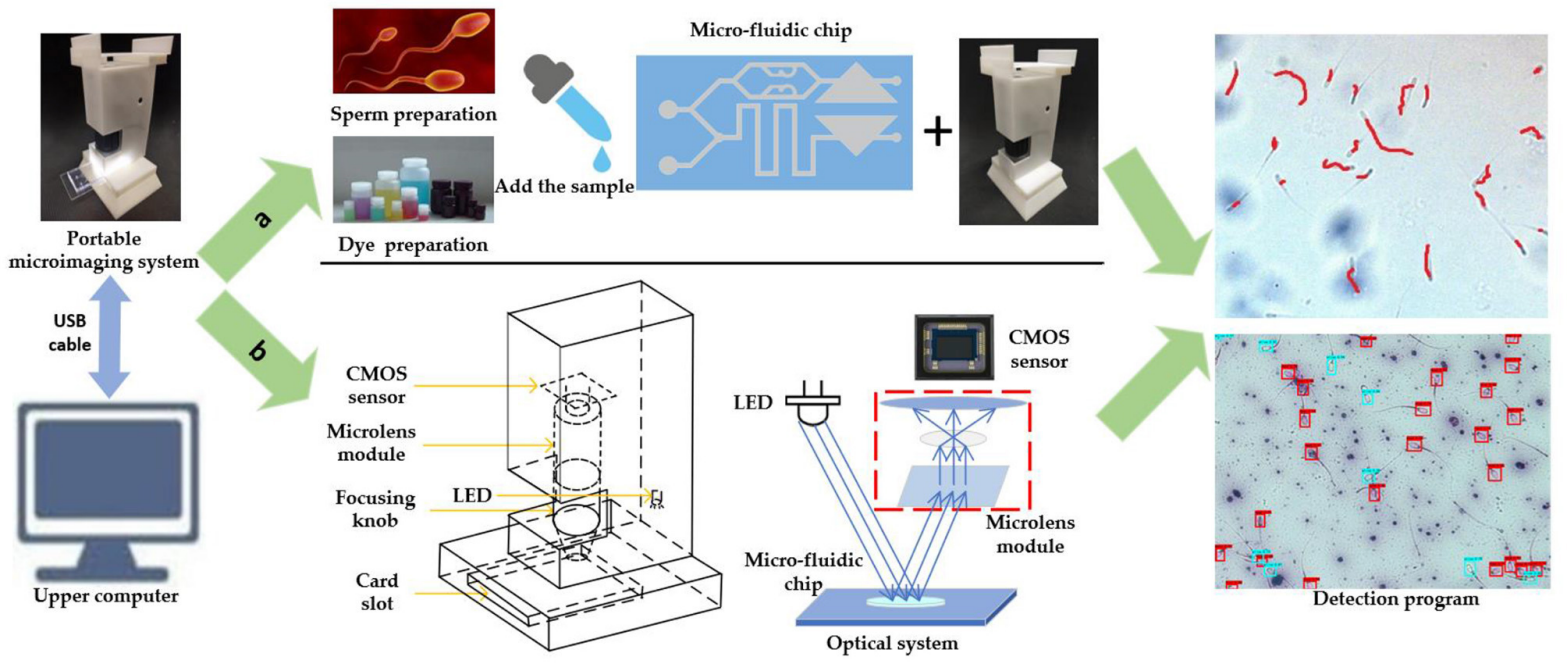
Schematic diagram of portable microscopic identification system. **(a)** Schematic diagram of system operation flow. **(b)** Schematic diagram of the structure of the portable microscope.

The schematic diagram of the portable micro-imaging recognition system is shown in Figure 2. It includes a portable micro-imaging equipment, a microfluidic chip and an upper computer. The portable micro-imaging device consists of a microlens array, an LED lamp and a CMOS image sensor. In order to realize the function of light amplification, the microfluidic chip is illuminated by the light from the LED. After the reflected light passes through the micro-lens group, the CMOS sensor receives the microscopic image. The device can achieve a 400 times magnification effect. The frame rate is 25 frames per second, and the spatial resolution is 2 μm per pixel. The recognition algorithm is executed in PyCharm 2020.2.3 under the Windows 10 operating system.

When the system is working, the configured sperm sample and dye are dropped into the corresponding inlet of the self-priming microfluidic chip. Then, the chip is inserted into the card slot of the imaging device, and then the microfluidic chip uniformly absorbs the liquid drops. You can fine tune the position of the chip for better observation. Because it takes a certain time for sperm to be stained completely, the sperm motility observation area is aligned with the CMOS sensor first, and the obtained image is transmitted to the host computer *via* USB cable. On the host computer, the sperm motility was identified with OpenCV. Finally, adjust the position of the chip until the sperm viability observation area is aligned with the CMOS sensor. Sperm survival rate identification is also performed in the upper computer. The comprehensive evaluation of sperm quality can be realized by the joint detection of sperm motility and survival rate.

### Algorithm Research of Portable Micro-Imaging Recognition System

The key to identifying sperm motility is to track sperm motility. The prerequisite of tracking is to separate the sperm. After obtaining the video of the sperm movement observation area, it is necessary to preprocess each frame image. The color of the sperm is darker than the background. The color image should be grayscaled first to obtain the grayscale image of the sperm image.


(1) $$\begin{array}{r} GRAY\;=\;0.229*\;R\;+\;0.587\;*\;G\;+\;0.114\;*\;B \end{array}$$

RGB is the three-channel color image, and gray is the single-channel grayscale image. The gray enhancement algorithm is used to widen the gap between sperm and background gray value, and improve the separation efficiency. The algorithm is expressed as:


(2) $$\begin{array}{r} H\left(x,y\right)\;=\;220\;*\;\left(G\left(x,y\right)\;-\;g_{\min}\right)/\left(g_{\max}\;-\;g_{\min}\right) \end{array}$$

where, *G*(*x,y*) is the gray value of the original gray image pixel; *g*_min_ is the minimum gray value of all pixels in the current frame; *g*_max_ is the maximum gray value of all pixels in the current frame; *H*(*x,y*) is the gray value after grayscale enhancement. So far, the preprocessing is completed. After that, an appropriate threshold is needed to distinguish between sperm and background. The adaptive threshold based on the Gaussian equation is expressed as:


(3) $$D(x,y)\;=\;\{\begin{array}{c} 1,|H(x,y)\;-\;g(x,y)|\;\geq T\\ 0,|H(x,y)\;-\;g(x,y)|\;<\;T \end{array}$$

where, *g*(*x,y*) is the Gaussian mean value of the pixel area after grayscale enhancement; T is the threshold value; and *D*(*x,y*) is the pixel value after thresholding. The threshold image is a finalized image, which contains information about sperm and a small amount of background noise. The connected area of the background noise is very small, and it can be filtered out by expansion and median filter. Therefore, the outline information of the sperm can be accurately obtained, and the position of the sperm centroid can be determined through the outline information.

After the sperm information is separated, the Kernelized Correlation Filters (KCF) is used to achieve multi-target tracking of sperm. Here, the Intersection over Union (IOU) is used as the matching standard. The calculation formula of IOU is as follows:


(4) $$\begin{array}{r} IOU\;=\;\frac{A\;\cap B}{A\;\cup B} \end{array}$$

**Figure 3:**
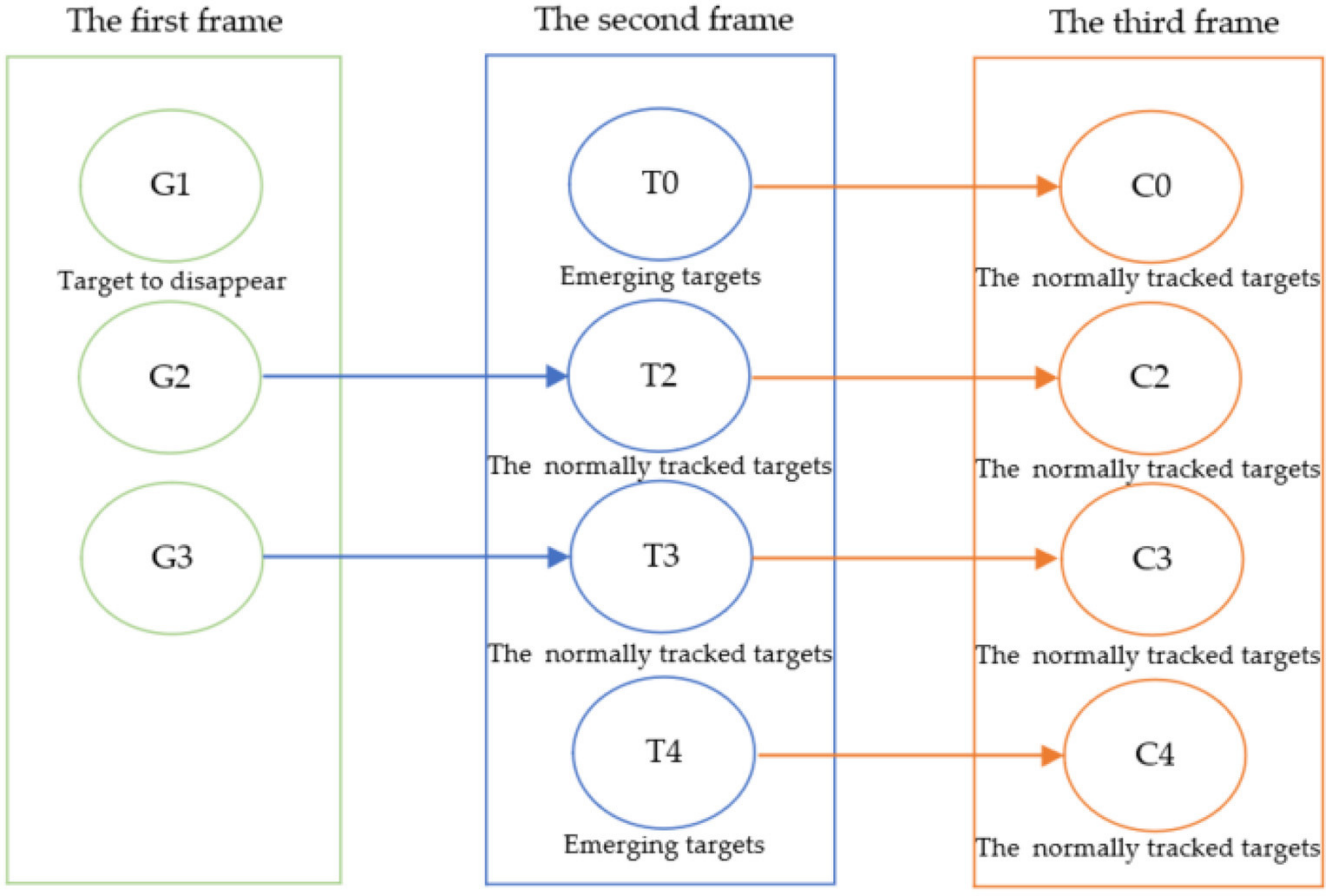
Schematic diagram of the multi-target association matching state of adjacent frames.

A refers to the predicted border area of the sperm by using KCF, and B refers to the actual border area of the sperm. If the IOU value of the predicted and the actual target areas are greater than 0.8 and the attributes are the same, the target will be associated with the target of the previous frame. During the association process of consecutive frames, there will be three situations, and the tracking strategy is shown in Figure 3:

1. Search and match the newly detected target in the tracked targets. If found, the target is successfully tracked. The detected target in the current frame and the previous frame are the same target;
2. If the newly detected target fails to be found in the tracked targets, indicating that the detected target is a new target. Record the target and generate a new tracker for tracking, which can be used for the next tracking association;
3. If no newly detected target is successfully associated with a tracked target, the target may disappear from the field of view and is currently in a lost state. Thus, its tracker needs to be deleted.

Sperm motility refers to the ability of the sperm to move forward to fertilize the oocyte. It is not only related to the speed of the sperm, but also related to whether the movement track of the sperm is close to a straight line. After the sperm is matched with the previous sperm, the center of the rectangular frame of the sperm outline is taken as the sperm center of mass. The actual distance between every two pixels in contact with each other is 2 μm. The movement parameters of sperm can be obtained by calculating the movement distance of the sperm centroid position.

Since the CMOS sensor collects 25 images in 1 s, 26 consecutive frames of images are used for estimation, dividing the sperm into four motility levels from A to D. Some researchers have used one or more CASA parameters to classify sperm movement (30–32). In this study, three CASA parameters are used to classify sperm motility, including VSL, VCL and linearity (LIN) to estimate the level of sperm motility. The formulas are expressed respectively as:


(5) $$\begin{array}{rcl} VSL_{i}\; & = & \;\frac{\left|\sqrt{{\left(x_{M}\;-\;x_{1}\right)}^{2}\;+\;{\left(y_{M}-y_{1}\right)}^{2}}\right|}{\left(M\;-\;1\right)\text{Δ}t} \end{array}$$

(6) $$\begin{array}{rcl} VCL_{i}\; & = & \;\frac{\sum_{j\;=\;1}^{M-1}|\sqrt{{\left(x_{j+1}\;-\;x_{j}\right)}^{2}\;+\;{\left(y_{j+1}\;-\;y_{j}\right)}^{2}}|}{\left(M-1\right)\text{Δ}t} \end{array}$$

(7) $$\begin{array}{rcl} LIN_{i}\; & = & \;\frac{VSL_{i}}{VCL_{i}} \end{array}$$

In formula (6), *x*_*j*_, *y*_*j*_ are the abscissa and ordinate of the pixel corresponding to the sperm center of the *j*-th frame; ▵*t* is the time interval between every two frames, which is 0.04 s here; *M* is the number of collected frames, which is 26 here; *VSL*_*i*_ is the ratio of the straight-line distance from the start position to the end position of the *i*-th sperm and the elapsed time; *VCL*_*i*_ is the ratio of the actual distance from the start position to the end position of the *i*-th sperm and the elapsed time; LIN is the linearity of the *i*-th sperm movement, defined as the ratio of *VSL*_*i*_ and *VCL*_*i*_. The standards for estimating sperm motility grades proposed in this paper are as shown in Table 1, which are based on World Health Organization (WHO) Semen Testing Laboratory Manual (33).

**Table 1:** Sperm motility grade estimation standard.

**Class**	**Description**	**Criteria VSL (μm/s) and VCL (μm/s) and LIN**
Class A	Sperm which swims rapidly in a straight line	VSL ≥ 24 and VCL ≥ 48 and LIN ≥ 0.5
Class B	Sperm which moves forward quickly, usually in a curved path	VSL ≥ 16 and VCL ≥ 40 and LIN ≥ 0.4
Class C	Sperm which moves slowly	REST
Class D	Sperm which barely moves	VSL ≤ 6 or VCL ≤ 16

*VSL, straight line velocity; VCL, curvilinear velocity; LIN, linearity*.

*REST: Sperm which doesn't belong to Class A, B and D*.

**Figure 4:**
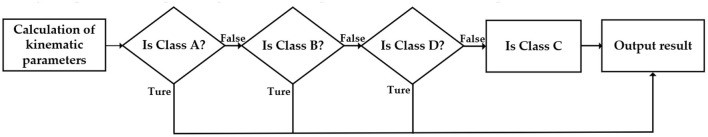
The logic of sperm motility level estimation. False means the estimation is wrong. Ture means the estimation is correct.

The logic of motion level estimation is shown in Figure 4. Firstly, estimate whether the sperm is Class A; if not, continue to estimate whether it meets the criteria of Class B; if not, turn to Class D; if still not, then estimate the sperm as Class C. Generally, Class A and Class B are considered as sperm which can be successfully fertilized, and the sperm motility is expressed as the percentage of Class A + B sperm to the total number of sperm.

**Figure 5:**
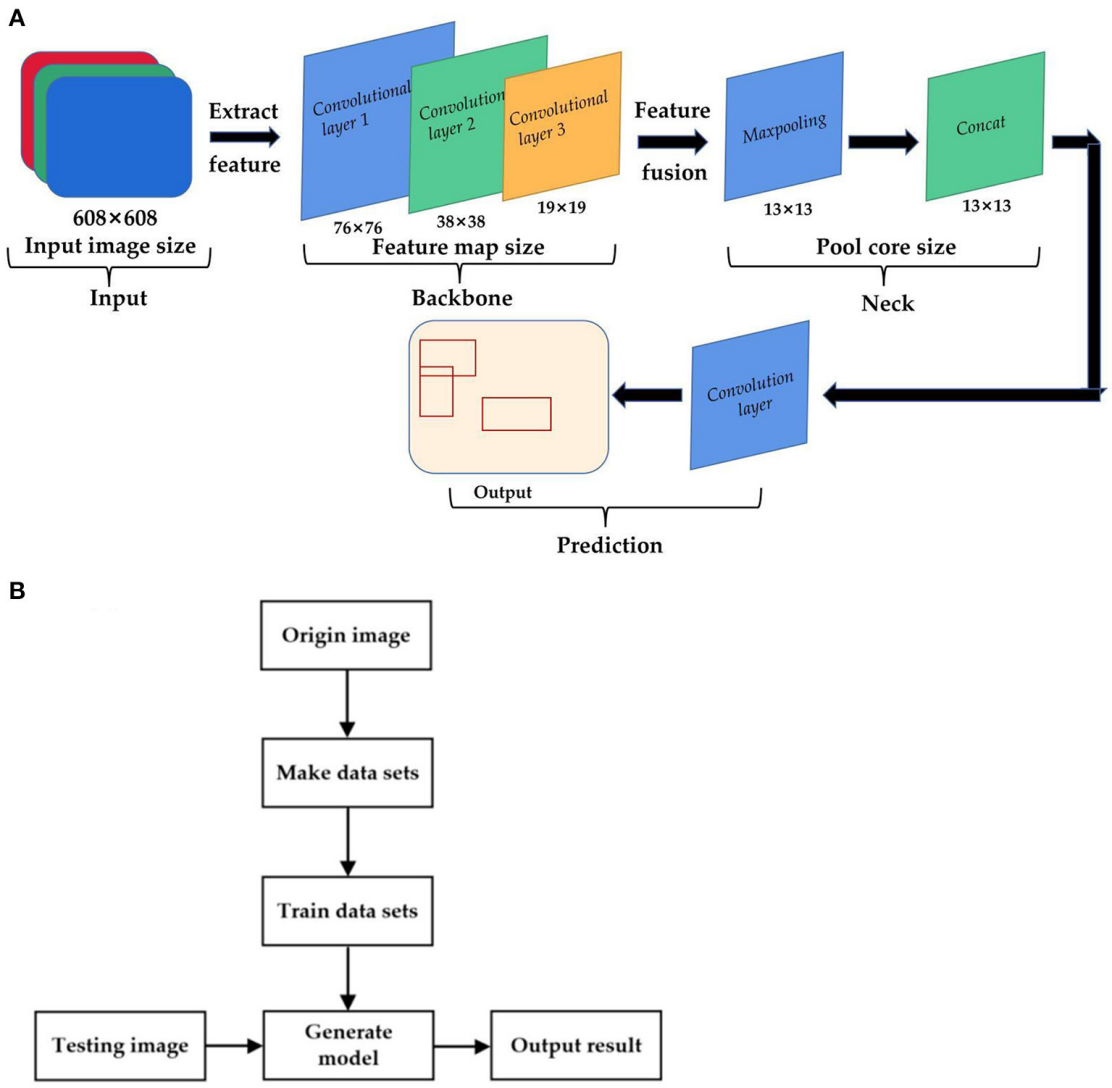
The principle of sperm survival detection based on YOLOv4. **(A)** YOLOv4 recognition model. **(B)** Flow chart of sperm survival rate detection based on YOLO4.

The dead sperm include inactive sperm and active sperm with membrane damage. Those sperm cann't resist foreign substances because the function of the membrane is incomplete. As a result, the staining solution can penetrate into the cell to stained cell. However, the head membrane of living sperm is not damaged, and the staining solution cann't penetrate, so the cells are not easy to be stained. Generally, after staining with eosin-aniline black, the head of live sperm is white or close to the background color, while the head of dead sperm is purple-red. In order to distinguish the dead sperm from the living sperm, YOLOv 4 is used to identify the sperm survival state. The principle of sperm survival detection based on YOLOv4 is shown in Figure 5. Firstly, the original image is randomly divided into the training set and verification set at a ratio of 9:1. Then the following steps are performed respectively. Make a data set: perform operations such as random rotation, cropping, and stretching on the original image, mark the survival status of the sperm at the same time. Train data set: put the marked data set into the model for training. Save the weight value of the YOLOv 4 model until the loss function value of this model does not change much. Output the result: Put the validation set into the trained model for sperm status detection, then output the recognition result. Sperm survival rate is equal to the ratio of the number of live sperm to the total number of sperm.

## Results and Discussion

### Self-Priming Microfluidic Chip

**Figure 6:**
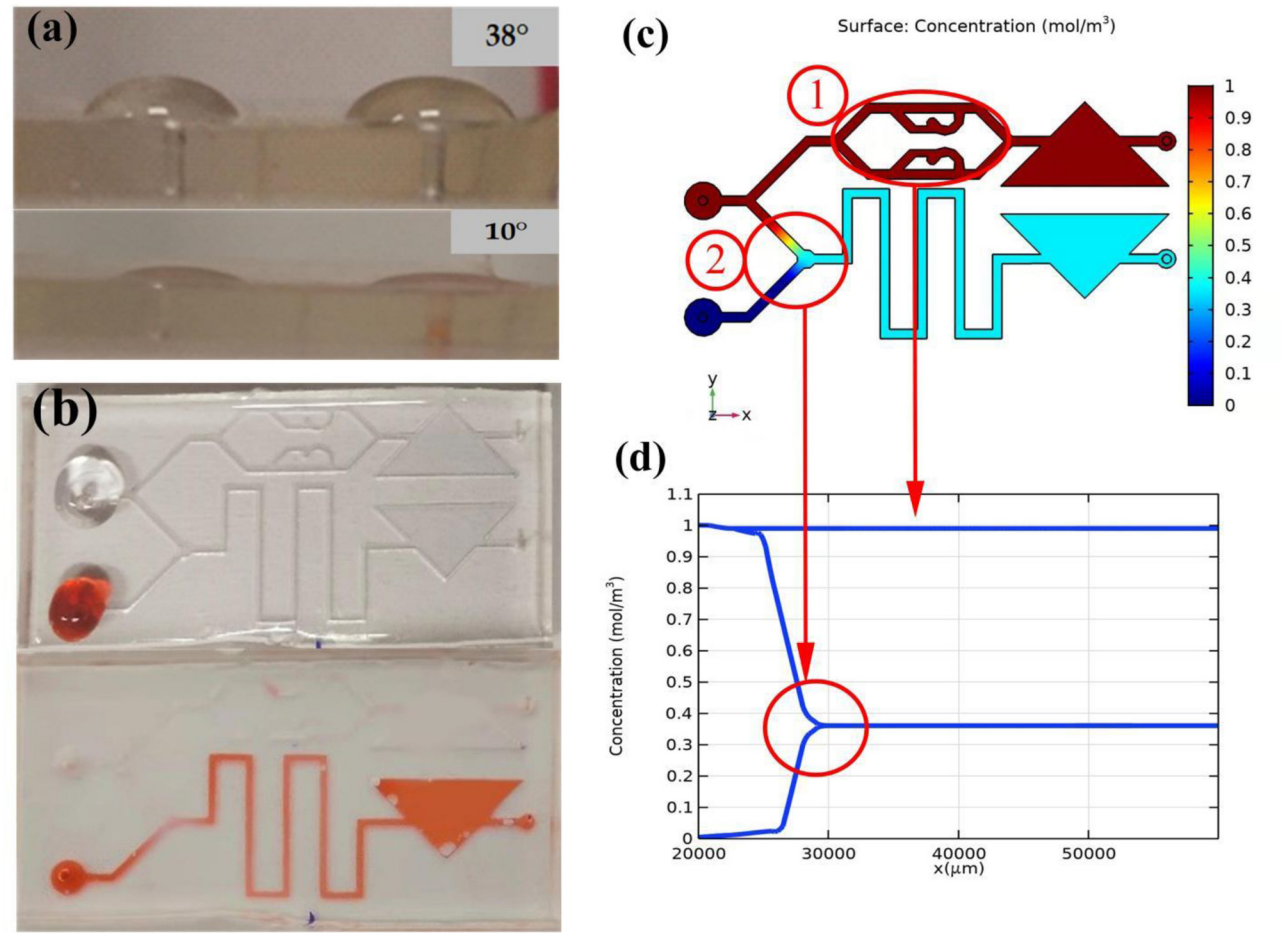
Self-absorption and mixing effect of the microfluidic chip. **(a)** Comparison of hydrophilic angle. **(b)** Self-priming effect comparison. **(c)** Mixed effects simulation. **(d)** Variation law of mixed concentration.

Due to the effect of the hydrophilic material, the contact angle of the surface of the water drop chip changes. Through measurement, the contact angle of the chip without treatment is about 38°, and the angle after treatment is about 10°, as shown in Figure 6a.

The structure of the microfluidic chip is simulated through COMSOL 5.5 to verify the mixing effect of the sample. The simulation result is shown in Figure 6. The different colors in b represent different concentrations. Two fluids of different concentrations are added to the two injection ports to represent the sperm sample and the stain respectively. The upper injection port flows into the sperm sample with a high initial solubility. In Figure 6c, Circle 1 represents the mixing effect in sample-mixing channel. Correspondingly, in Figure 6d, when sperm sample flows through the sample-mixing channel, its concentration remains unchanged. Circle 2 represents the mixing effect in mixing area. The upper injection port flows into the dye with a low initial solubility. In the sample mixing area, the dye is completely mixed with the sperm sample, and the concentration difference decreases rapidly until zero.

In order to test the self-aspiration and mixing effect of the chip, we use two pipettes (0.5–10 μl, eosin-aniline black; 0.5–20 μl, sperm sample) to simultaneously add two samples to the corresponding injection ports. As shown in Figure 6b, the untreated chip retains liquid at the entrance and the liquid is difficult to flow into the chip without external force, while the processed chip can quickly and automatically suck liquid. In the mixing zone, the sperm sample and stain were successfully mixed, because the color of the liquid in the buffer channel became lighter.

### Sperm Recognition

**Figure 7:**
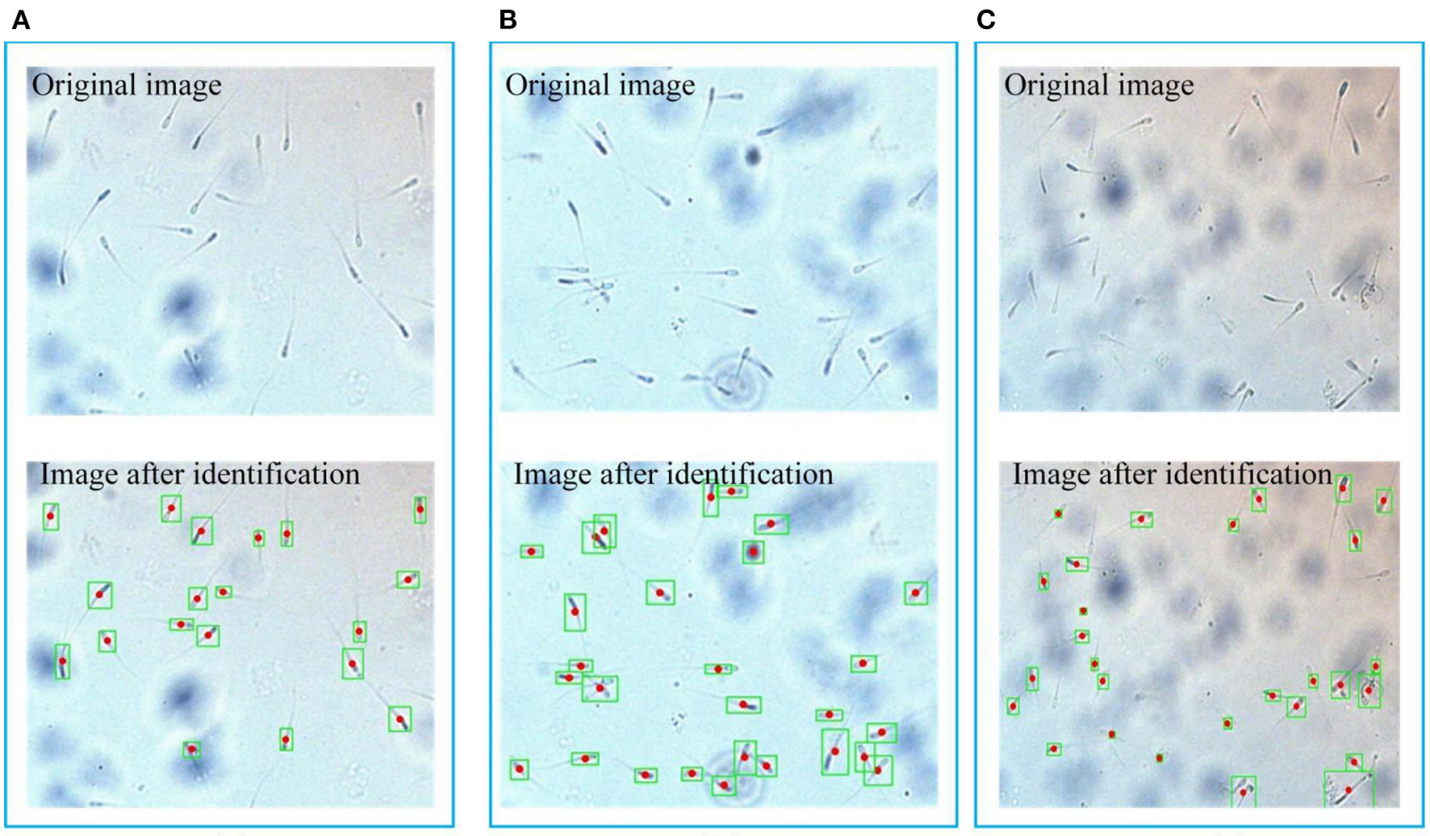
Boundary and centroid of the sperm. **(A)**, **(B)** and **(C)** respectively represent three groups of original images and processed images, the green box is the boundary of the sperm, and the red dot is the sperm center of mass.

When the sample flows into the observation area, stop the injection. The background flow velocity is zero. The motion observation area of the microfluidic chip is aimed at the camera, and the video of sperm motion is obtained at first. After the basic pre-processing of each frame of the image, the sperm and the background have a significant difference in gray value. Adaptive threshold processing is used to separate the sperm, but the processed image still contain some background noise. These background noises usually have small connecting areas. They have been filtered out mostly after expansion and median filtering. After filtering, only the information of the sperm is left. The boundary and centroid of the sperm are shown in Figure 7. Five sperm samples are tested and each sample takes three pictures. It can be seen that the sperm is well recognized, and the center of mass is mainly marked on the neck of the sperm. The results of sperm recognition are shown in Table 2. The accuracy rate is equal to the ratio of the number of identified sperm to the actual number of sperm, which is 95.6%. The error rate is equal to the ratio of the number of wrongly identified sperm to the actual number of sperm, which is 6.0%. Therefore, the image processing and recognition algorithm can achieve accurate sperm recognition. It can be seen from Table 2 that we have conducted five batches of experiments, and the identification accuracy of sperm samples has a certain difference. The source of the difference may be due to the following three reasons. First, the sperm samples in the five batches may contain impurities. When using the algorithm to identify the sperm, the algorithm will mistake the impurities as sperm. Second, the dilution and deactivation of sperm in this study may lead to the death of some sperm, and the dead sperm may have adhesions. When the algorithm is used to identify sperm, the adhesion of sperm may be mistaken for a sperm. Third, the sperm may overlap or stick together during the movement process, and certain errors may occur when using the algorithm to identify the sperm. However, from the test results of five batches, it can be seen that the recognition accuracy of the algorithm proposed in this study is still reliable.

**Table 2:** Results of sperm recognition.

**Sample label**	**Picture1**			**Picture2**			**Picture3**		
	**Identified number**	**Actual number**	**Precision**	**Identified number**	**Actual number**	**Precision**	**Identified number**	**Actual number**	**Precision**
Sample1	20	19	100%	19	18	100%	21	20	100%
Sample2	28	29	89.7%	22	21	95.2%	25	25	88%
Sample3	33	31	100%	29	30	90%	29	30	90%
Sample4	21	21	95.2%	18	18	94.4%	23	23	95.7%
Sample5	28	27	100%	30	29	100%	25	25	96%

### Trajectory Tracking of Sperm Targets

**Figure 8:**
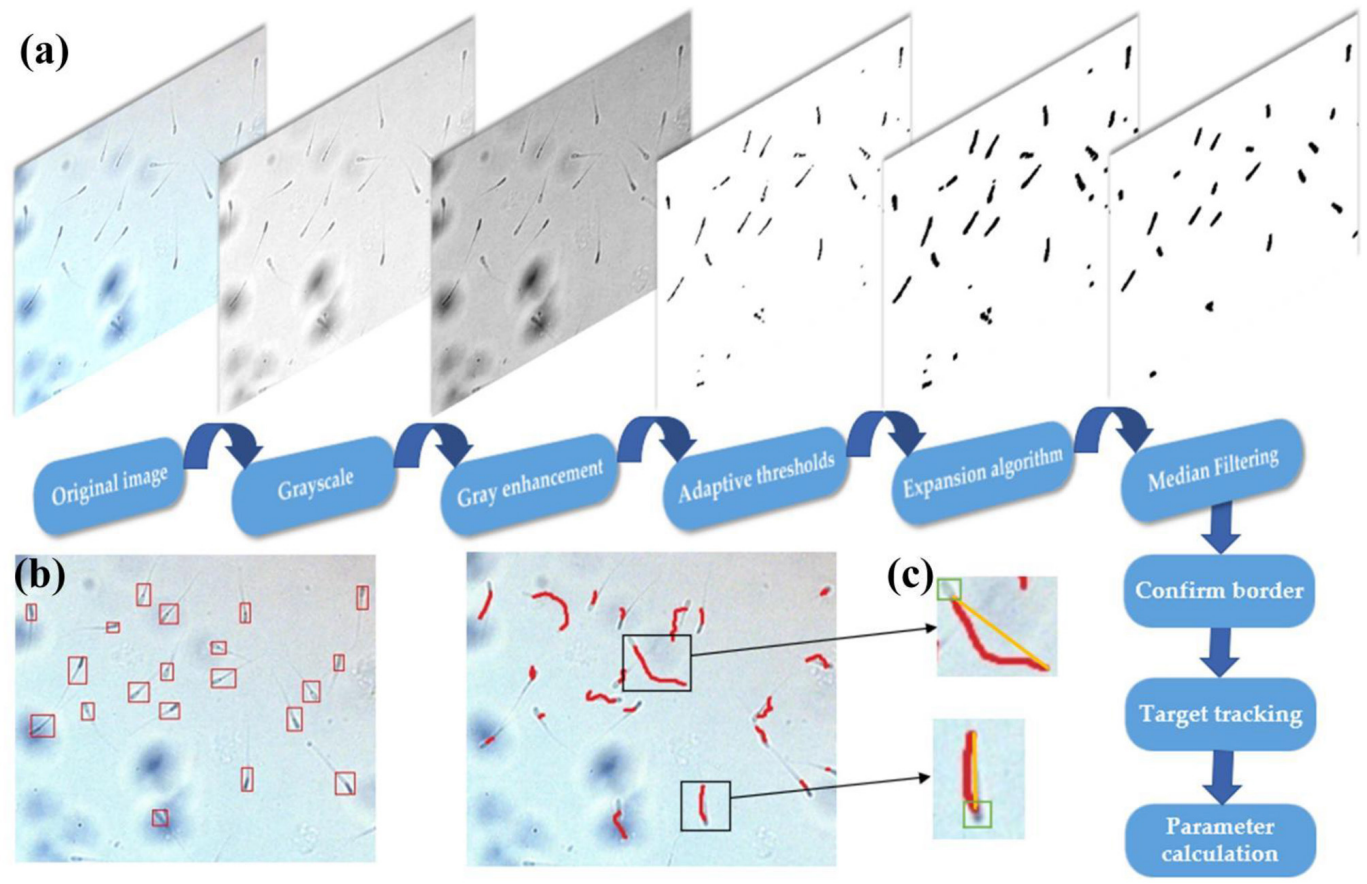
Sperm motility detection process. **(a)** Sperm image pre-processing. **(b)** Sperm multi-target tracking trajectory. **(c)** Single sperm target tracking trajectory (the yellow line is the linear motion trajectory of the sperm target, and the red line is the actual motion trajectory).

The separated sperm image is a binary image. The background information has been removed, and only useful contour information is retained. The method of KCF combined with IOU is used to perform multi-target matching and tracking of the sperm. The relevant parameters are determined according to the change of the centroid position. The movement trajectory of sperm was tracked by Minitube Sperm Vision automatic sperm analyzer (Beijing Bluader Technology Development Co., LTD, Beijing, China). Figure 8 shows the process of sperm motility detection process.

**Figure 9:**
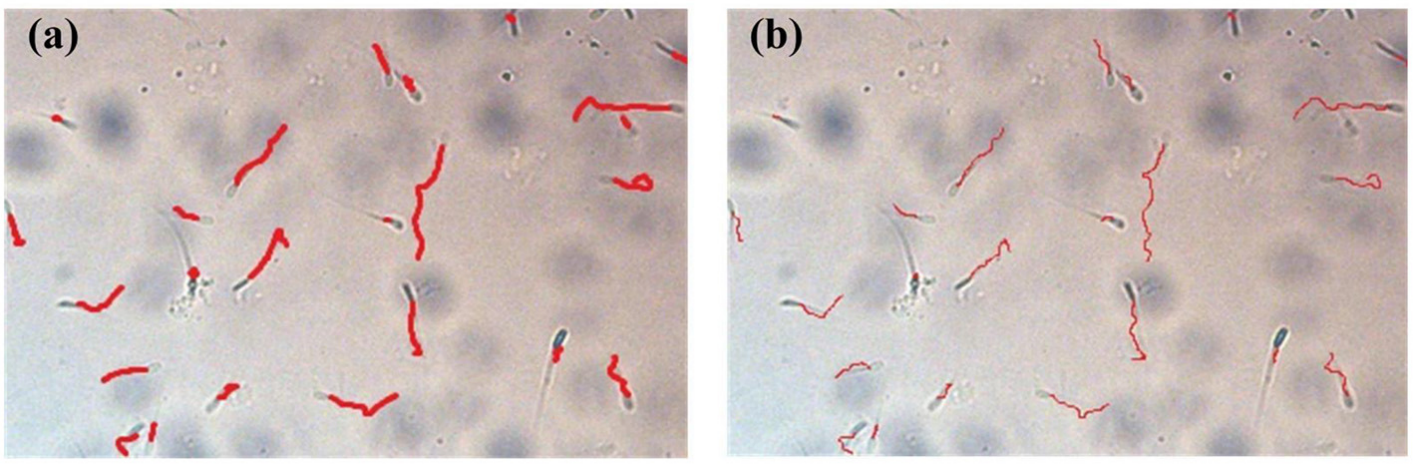
Comparison between sperm trajectory tracking based on OpenCV and CASA. **(a)** Sperm trajectory tracking based on OpenCV. **(b)** Sperm trajectory tracking based on CASA.

Taking the boundary position of the sperm in the first frame as the initial position, the multi-target tracking of the sperm is achieved through KCF and IOU. At the same time of OpenCV detection, the video of sperm movement is saved, and the video is estimated by CASA. Figure 9 shows comparison between sperm trajectory tracking based on OpenCV and CASA. The trajectory tracked by OpenCV is close to that tracked by CASA. Therefore, the proposed algorithm can track sperm well.

### Estimation of Sperm Motility

**Figure 10:**
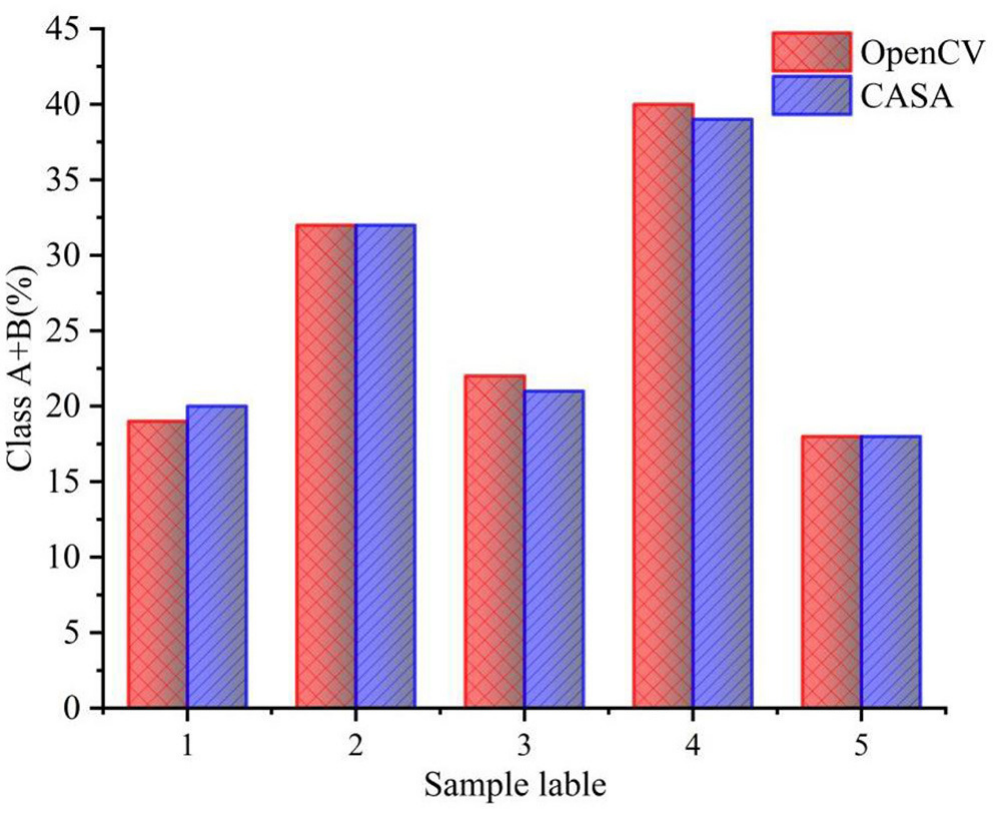
Comparison between sperm motility estimation based on OpenCV and CASA.

straight line velocity, VCL and LIN are used to comprehensively estimate the motility level. After determining the motility level of each sperm, the overall motility of the sperm is represented by the percentages of Class A + B to the total number of sperm. Table 3 shows the results of the overall sperm motility level estimation by OpenCV. The motility of sperm samples is about 30%. As can be seen from Table 3, although the algorithm proposed in this study can make a good judgment on the sperm motility, it can be seen from the judgment results of the sperm motility of the five samples that the sperm motility of the five samples has a certain degree. difference. The source of the difference in sperm motility may be due to the dilution and deactivation of sperm in this study before the experiment. Different batches of sperm will have different viability after dilution and deactivation treatment. Figure 10 shows the comparison between sperm motility estimation based on OpenCV and CASA. The results of OpenCV and CASA are very close, as *r* is equal to 0.997. As a result, the sperm motility standard and detection are feasible.

**Table 3:** Results of the overall sperm motility level estimation by OpenCV.

**Percentage**	**Class A**	**Class B**	**Class C**	**Class D**	**Class A + B**
Sample1	8%	11%	52%	29%	19%
Sample2	16%	16%	46%	22%	32%
Sample3	11%	11%	50%	28%	22%
Sample4	22%	18%	36%	24%	40%
Sample5	9%	9%	55%	27%	18%

### Estimation of Sperm Survival Rate

**Figure 11:**
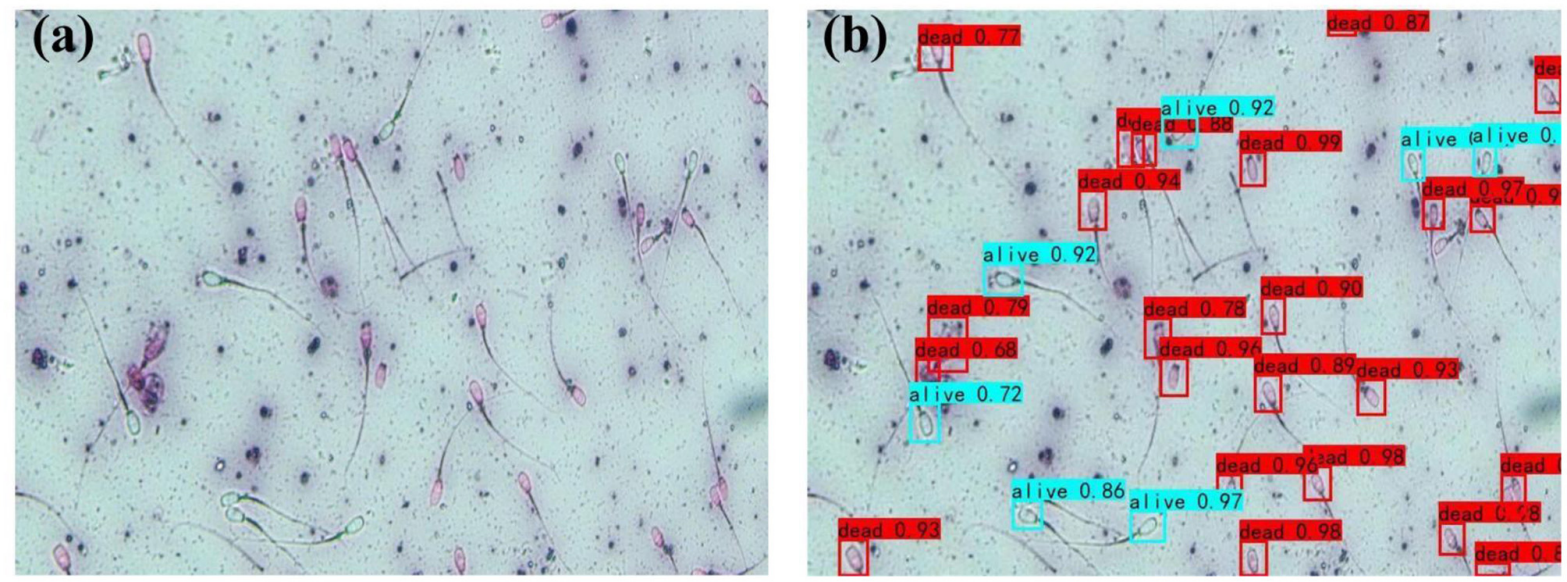
YOLO4 recognition results. **(a)** Original image. **(b)** Image after testing.

After estimating the sperm motility, the microfluidic chip is moved until the sperm survival rate observation area is aligned with the camera to obtain the image of the stained sperm. YOLOv4 will first identify the sperm in the image, and then determine the credibility that the identified sperm is dead sperm. The credibility range is [0,1]. The sperm were divided according to their activity observed artificially under a microscope. That is, if the credibility is ≥0.5, it will be estimated as dead sperm. On the contrary, if the credibility is <0.5, it is estimated as live sperm. The recognition results are shown in Figure 11, the red box indicates that the sperm is identified as dead sperm, and the blue box indicates that it is identified as live sperm. The recognition results of YOLOv4 are compared with the results of the observer. Through analyzing the collected image data, the accuracy of the YOLO4 recognition algorithm is 94.0%, and the recognition error is 0.6%. In clinical medicine, inaccurate estimations are inevitable. As long as the difference between the number of surviving sperm identified and the total number of sperm is very small, the algorithm can still accurately identify the survival rate of the sperm.

In order to further illustrate the reliability of the results of this study, comparative analysis was conducted between the sperm survival rate identified in this study and the actual sperm survival rate. The results are shown in Table 4. It can be seen from Table 4 that a total of five sets of experiments were carried out, with an error range of the experiment was 1.44%−13.73%, and the average error of the experiment was 7.16%. Although there is some error between the sperm survival rate identified in this study and the actual sperm survival rate, the overall accuracy is still trustworthy and falls within the acceptable range (34).

**Table 4:** Results of identified and actual survival rate (%).

**Sample**	**Identified survival rate**	**Actual survival rate**	**Error**	**Average error**
1	25	23.3	7.29	7.16
2	26.5	23.3	13.73	
3	26.7	25	6.8	
4	28.2	27.8	1.44	
5	20	21.4	6.54	

### Comparison of Methods

The system designed in this paper can achieve a comprehensive estimation of sperm quality by integrating sperm motility and survival rate detection on a microfluidic chip. As we can known form Table 5, compared with Routine Sperm Analysis (RSA), it can greatly reduce the operation steps, improve the detection efficiency, and avoid the subjectivity of manual observation. Compared with Computer Assisted Sperm Analysis (CASA), it can estimate the survival rate of sperm while maintaining the accuracy of motility detection. The combined detection of survival rate and motility can more accurately reflect sperm quality. Compared with home-based sperm analysis, method in this paper can be used for quantitative measurement of sperm motility. Besides, most home-based sperm analysis can't measure survival rate. In addition, although the method proposed in this study can detect sperm well, it also has certain defects. For example, the system needs to be completed by means of a microfluidic chip, and the repeatability of the microfluidic chip itself is poor, and the current manufacturing process is relatively complicated (35). Of course, with the development of micro-nano manufacturing technology in the future, this shortcoming will be gradually overcome.

**Table 5:** Comparison of methods.

**Methods**	**Detection accuracy**	**Detection time**	**Survival rate**	**Motility**
Routine sperm analysis	^*^	^*^	**√**	**√**
Computer assisted sperm analysis	97%	5s	**×**	**√**
Home-based sperm analysis	Qualitative analysis	**–**	**×**	**√**
Method in this paper	94%	9s	**√**	**√**

* *The accuracy and time of detection are related to oberver's experience*.

*–Different home-based products require different detection time (36)*.

## Conclusions

In this paper, a portable micro-imaging system based on a microfluidic chip was designed. This device can accurately identify stained and unstained sperm, as well as quickly determine the sperm motility level. With the relevant algorithms of OpenCV, it can accurately estimate sperm survival rate and track movement trajectory. The survival rate identification accuracy of the algorithm is 94.0%, and the estimation of the sperm motility level is consistent with that of CASA. In general, applying this method for comprehensive estimation of sperm quality through the detection of sperm motility and survival rate is feasible. However, due to long exposure time of the captured pictures, some images have afterimages, which increases the discrimination error to a certain extent. Therefore, the algorithms need to be optimized in the future.

## Data Availability Statement

The original contributions presented in the study are included in the article/supplementary material, further inquiries can be directed to the corresponding author.

## Author Contributions

Conceptualization and methodology: NY. Formal analysis: YW. Data curation: XP and KG. Writing—original draft preparation: KG and NY. Writing—review and editing: XP, KG, XW, XZ, LS, PZ, FQ, JY, and YW. All authors have read and agreed to the published version of the manuscript.

## Funding

This research was funded by Jiangsu Agricultural Science and Technology Innovation Fund (CX (20)2013), China Agriculture Research System of MOF and MARA (CARS-43-G-2), Project of Agricultural Equipment Department of Jiangsu University (NZXB20210106 and NZXB20200205), Zhenjiang Key R&D Program-Modern Agriculture (NY2019013), and Zhenjiang Key R&D Program-Social Development (SH2019012).

## Conflict of Interest

The authors declare that the research was conducted in the absence of any commercial or financial relationships that could be construed as a potential conflict of interest.

## Publisher's Note

All claims expressed in this article are solely those of the authors and do not necessarily represent those of their affiliated organizations, or those of the publisher, the editors and the reviewers. Any product that may be evaluated in this article, or claim that may be made by its manufacturer, is not guaranteed or endorsed by the publisher.
